# Hepatoma polarization limits CD81 and hepatitis C virus dynamics

**DOI:** 10.1111/cmi.12047

**Published:** 2012-11-20

**Authors:** H J Harris, C Clerte, M J Farquhar, M Goodall, K Hu, P Rassam, P Dosset, G K Wilson, P Balfe, S C IJzendoorn, P E Milhiet, J A McKeating

**Affiliations:** 1 School of Immunity and Infection, University of BirminghamBirmingham, UK; 2 NIHR Centre for Liver Disease, University of BirminghamBirmingham, UK; 3 Unité 1054, InsermMontpellier, France; 4 Centre de Biochimie Structurale, Université de Montpellier, CNRS, UMR 5048Montpellier, France; 5 Department of Cell Biology, University Medical Center Groningen, University of GroningenGroningen, The Netherlands; 6 NIHR Liver Biomedical Research Unit, University of BirminghamBirmingham, UK

## Abstract

Many viruses target the polarized epithelial apex during host invasion. In contrast, hepatitis C virus (HCV) engages receptors at the basal surface of hepatocytes in the polarized liver parenchyma. Hepatocyte polarization limits HCV entry by undefined mechanism(s). Given the recent reports highlighting a role for receptor mobility in pathogen entry, we studied the effect(s) of hepatocyte polarization on viral receptor and HCV pseudoparticle (HCVpp) dynamics using real-time fluorescence recovery after photobleaching and single particle tracking. Hepatoma polarization reduced CD81 and HCVpp dynamics at the basal membrane. Since cell polarization is accompanied by changes in the actin cytoskeleton and CD81 links to actin via its C-terminus, we studied the dynamics of a mutant CD81 lacking a C-terminal tail (CD81_ΔC_) and its effect(s) on HCVpp mobility and infection. CD81_ΔC_ showed an increased frequency of confined trajectories and a reduction of Brownian diffusing molecules compared to wild-type protein in non-polarized cells. However, these changes were notobserved in polarized cells. HCVpp showed a significant reduction in Brownian diffusion and infection of CD81_ΔC_ expressing non-polarized cells. In summary, these data highlight the dynamic nature of CD81 and demonstrate a role for CD81 lateral diffusion to regulate HCV infection in a polarization-dependent manner.

## Introduction

Hepatitis C virus (HCV) is a member of the Flaviviridae family and an important human pathogen that infects hepatocytes leading to progressive liver disease including fibrosis, cirrhosis and hepatocellular carcinoma. At present there is no HCV vaccine and there is an international effort to develop new antiviral agents. A number of drugs targeting HCV replicase enzymes are in development; however, recent trials show a rapid appearance of drug-resistant viruses (Asselah and Marcellin, 2011; Pawlotsky, 2011). The essential and conserved nature of HCV entry into host cells offers an attractive target for therapeutic intervention. HCV initiates infection by attaching to molecules or receptors at the cell surface. Current evidence supports a role for scavenger receptor class B member I (SR-BI), tetraspanin CD81 and tight junction proteins claudin-1 and occludin in co-ordinating a clathrin-dependent endocytic internalization of HCV; however, the role of individual receptors in this process is unclear (reviewed in Meredith *et al*., 2012).

SR-BI and CD81 bind HCV-encoded E1E2 glycoproteins with high affinity (Pileri *et al*., 1998; Scarselli *et al*., 2002), suggesting a classical role in defining particle attachment to the cell surface. In contrast, there is limited information demonstrating claudin-1 or occludin interaction with HCV, suggesting an indirect role for these proteins in particle entry. SR-BI is the major receptor for high-density lipoprotein (Krieger, 2001) and mediates the trafficking of cholesterol to and from lipoproteins by selective cholesterol uptake and cholesterol efflux (Acton *et al*., 1996; Rhainds and Brissette, 2004; Nieland *et al*., 2005). CD81 is a member of the tetraspanin family that has diverse roles in cell–cell adhesion, cell migration and immune cell function (Hemler, 2005). One distinctive feature of this protein family is their propensity to associate with other tetraspanin and non-tetraspanin proteins to form multimolecular complexes or tetraspanin-enriched areas or microdomains (TEAs or TEMs) (Hemler, 2005; Levy and Shoham, 2005). CD81 links to the cytoskeleton via its C-terminal association with phosphorylated ezrin (Sala-Valdes *et al*., 2006; Coffey *et al*., 2009), a member of the ezrin–radixin–moesin (ERM) family of proteins, providing a mechanism for HCV to access the actin cytoskeleton and promote virus internalization. Our recent observation that HCV increases CD81 endocytosis and fusion with Rab5 expressing early endosomes supports a role for CD81 in clathrin-dependent internalization of HCV (Farquhar *et al*., 2012).

Hepatocytes in the liver are polarized with a basal surface facing the circulation and a branched network of grooves between adjacent cells constituting the apical or bile canalicular surface (Decaens *et al*., 2008). HCV entering the liver via the sinusoidal blood will initially encounter receptors expressed at the basal surface of the hepatocyte. This is in contrast to airway pathogens (rhinovirus, respiratory syncytial virus and influenza) or enteric viruses (enteroviruses and rotaviruses) that infect polarized epithelia lining the lung airways or gastrointestinal tract, respectively, via the apical membrane that is known to restrict the movement of pathogens and macromolecules across mucosa (reviewed in Bergelson, 2009; Delorme-Axford and Coyne, 2011). We previously reported that hepatoma polarization restricts HCV entry (Mee *et al*., 2009), suggesting that the reduced accessibility of apical located tight junction proteins contributes to the limited infection of polarized cells. However, recent reports from our laboratory showing that non-junctional pools of claudin-1 associate with CD81 at the basal membrane challenge this model and raise questions as to the mechanism(s) restricting infection of polarized hepatocytes (Harris *et al*., 2008; 2010). Thus, the molecular mechanism(s) Coxsackie B virus and adenovirus have evolved to infect polarized epithelia via the apical surface (Coyne and Bergelson, 2006; Lutschg *et al*., 2011) may not be the most appropriate models for HCV or other liver tropic pathogens that target the basal hepatocellular membrane.

Advances in imaging technologies to track virus particles demonstrate an essential role for receptor mobility in pathogen entry (Mellman and Nelson, 2008; Burckhardt and Greber, 2009). To ascertain a role for SR-BI and CD81 dynamics in HCV infection, we used fluorescence recovery after photobleaching (FRAP) and single particle tracking (SPT) techniques to quantify receptor and HCV pseudoparticle (HCVpp) diffusion in polarized and non-polarized hepatocytes. Our real-time imaging studies show reduced lipid, CD81 and HCVpp dynamics at the basal membrane of polarized HepG2 cells compared to non-polarized cells. Furthermore, we show a role for the CD81 C-terminus in defining protein mobility and HCVpp diffusion. In summary, this study highlights the dynamic nature of CD81 and demonstrates a role for CD81 lateral diffusion in HCV infection of hepatoma cells that is dependent on their polarized status.

## Results

### Fluidity of the basolateral membrane

**Fig. 1:**
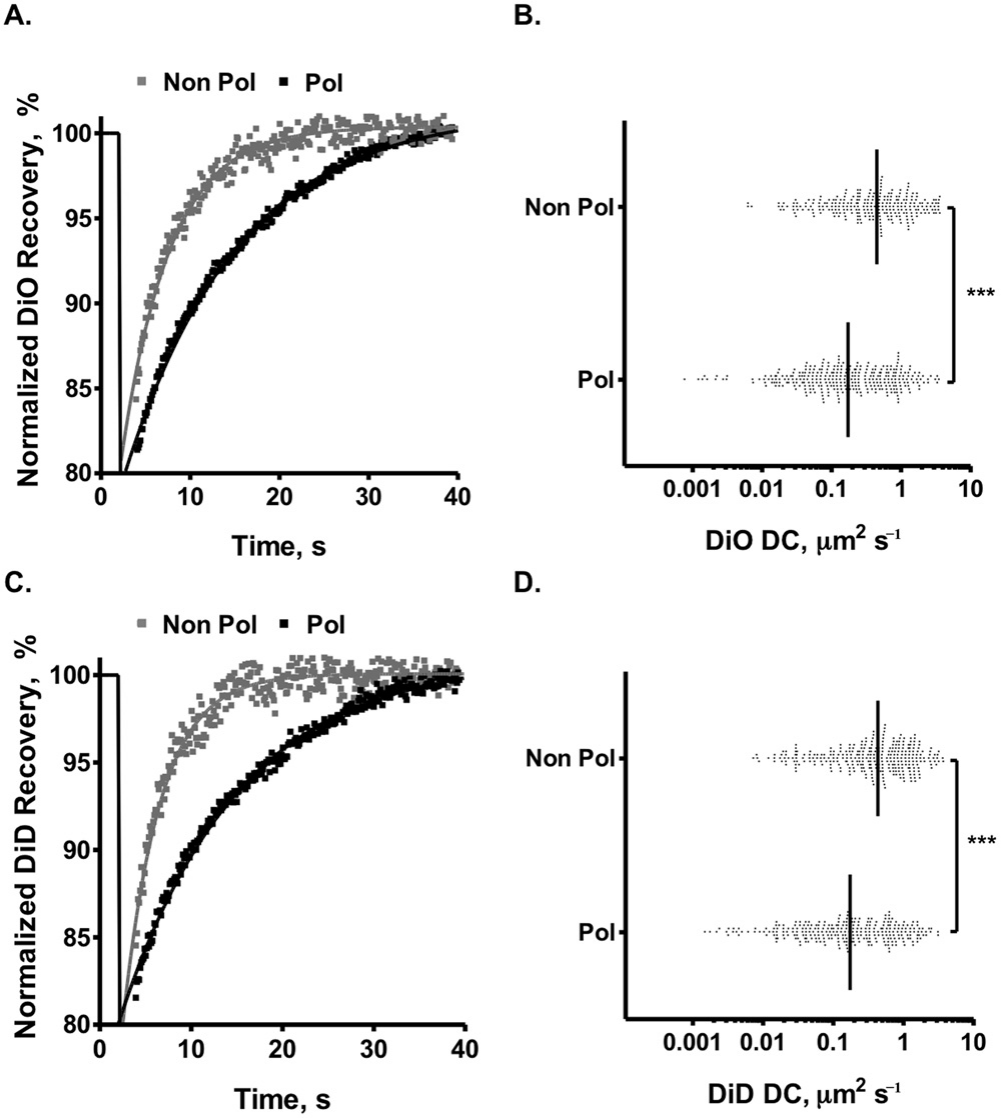
Effect of HepG2 polarization on lipid dynamics. Quantitative FRAP measurement of DiO-C18 and DiD-C18 dynamics in polarized (day 7 post plating with a polarization index of 43%) and non-polarized HepG2 cells (day 1 post plating with a polarization index of 2%). Representative normalized FRAP recovery curves in polarized (Pol, black) and non-polarized (Non Pol, grey) cells are shown. Median diffusion coefficient (DC) of DiO-C18 (A,B) and DiD-C18 (C,D) in polarized and non-polarized HepG2 cells, where each point represents one bleached region of interest and the vertical black line represents the median value. Mobile fraction of DiO (polarized – 92.7%, non-polarized – 86.4%; *P* < 0.001) and DiD (polarized – 91.5%, non-polarized – 86.4%; *P* < 0.001). A minimum of 10 cells and 100 ROIs were measured per parameter. Non-parametric Mann–Whitney *t*-tests were used to determine the degree of significance (*P < 0.05, **P < 0.001, ***P < 0.0001).

We previously reported that hepatoma polarization limits HCV entry (Mee *et al*., 2009); however, the mechanism underlying this restriction is unknown. The HepG2 hepatoma cell line is a well-characterized model system that polarizes over time and develops apical lumen that represents bile canaliculae (Ohgaki *et al*., 2010). Polarization is accompanied by a segregation of plasma membrane lipids and proteins to apical and basal membranes. Since membrane fluidity or viscosity is affected by many factors including lipid composition, we investigated potential differences in lipid mobility between polarized and non-polarized HepG2 membranes using fluorescent lipid probes. We used FRAP to quantify the apparent diffusion coefficient (DC) of fluorescent lipophilic carbocyanine dyes DiD-C18 and DiO-C18. These lipids incorporate into cell membranes and diffuse according to the lamellar order of the membrane (Klausner and Wolf, 1980; Ladha *et al*., 1997). Normalized FRAP recovery curves for DiO and DiD are shown in Fig. 1 that take into account background fluctuations and the effect of photobleaching during image acquisition. Rapid FRAP measurements (0.08 s per frame) show a significantly slower diffusion of both lipids in polarized HepG2 cells (DC = 0.17 μm^2^ s^−1^) compared to non-polarized cells (DC = 0.43–0.45 μm^2^ s^−1^) (Fig. 1). This change in membrane environment may contribute to the reduced HCVpp entry observed in polarized cells.

### Hepatitis C virus receptor mobility on polarized HepG2 cells

**Fig. 2:**
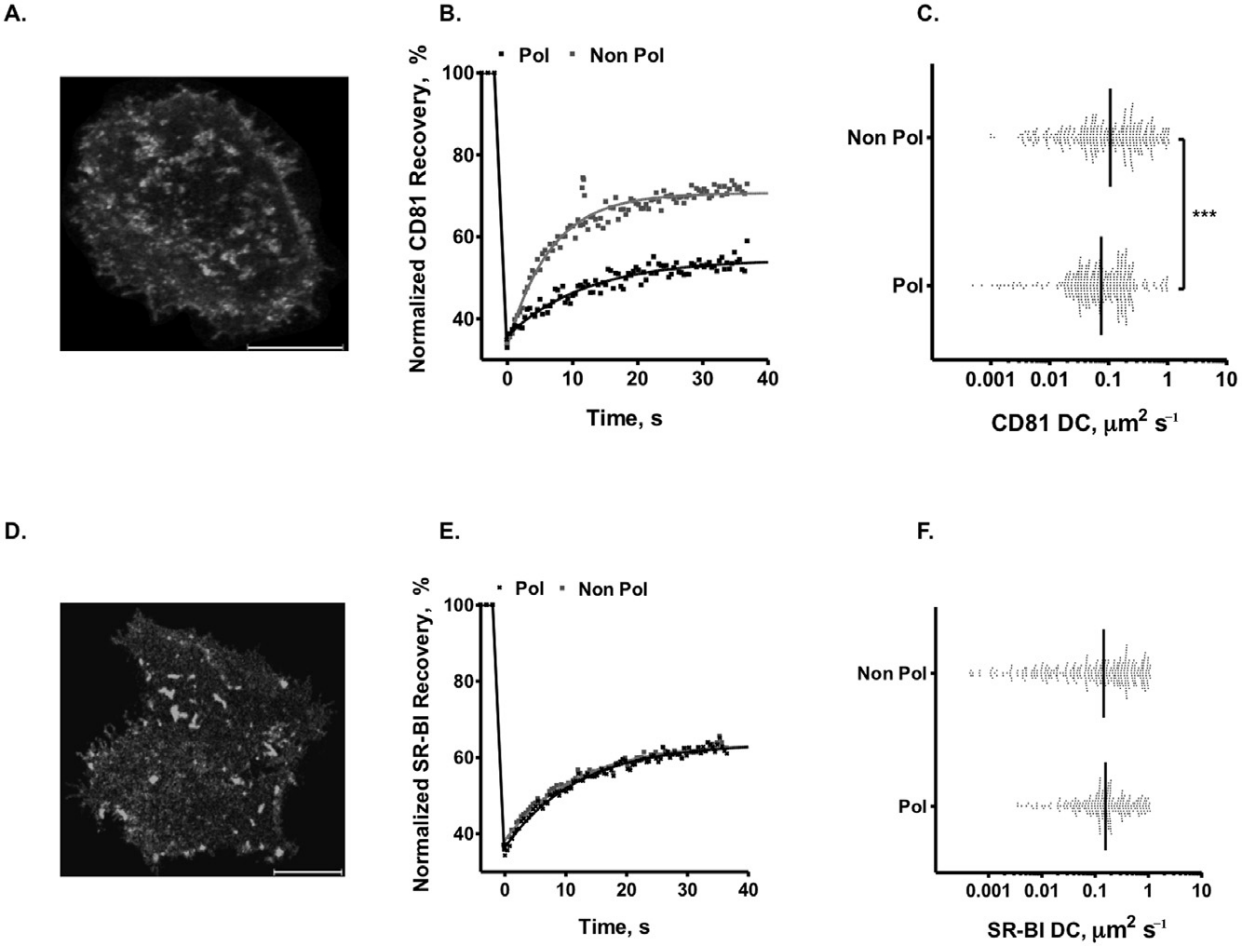
Effect of HepG2 polarization on CD81 and SR-BI dynamics. HepG2 cells were transfected to express AcGFP–CD81 (A) and AcGFP–SR-BI (D), and representative confocal images of non-polarized HepG2 cells (day 1 post plating with a polarization index of 2%) taken at the cell–glass interface are shown. The images were acquired using a 100× 1.4NA objective on the Zeiss LSM 780 confocal with 0.44 μm Z sections. Representative normalized FRAP recovery curves for CD81 (B) and SR-BI (E) in polarized (Pol, black) and non-polarized (Non Pol, grey) cells. Mobile fraction (*Mf*) of CD81 (polarized – 50.4 ± 19%, non-polarized – 67.7 ± 18%; *P* < 0.001) and SR-BI (polarized – 61.5 ± 17%, non-polarized – 58.3 ± 16%; *P* < 0.001). The diffusion coefficient (DC) for CD81 (C) and SR-BI (F) in polarized and non-polarized HepG2 cells is shown, where each point represents a bleached region of interest and the vertical black line represents the median value. A minimum of 10 cells and 100 ROIs were measured per parameter. Non-parametric Mann–Whitney *t*-tests were used to determine the degree of significance (*P < 0.05, **P < 0.001, ***P < 0.0001).

Recent studies have highlighted the importance of host cell transmembrane receptor dynamics in regulating virus particle trafficking (Burckhardt and Greber, 2009). We therefore studied the effect of HepG2 polarization on the dynamics of HCV ‘receptors’ CD81 and SR-BI. HepG2 cells were transduced to express *Aequorea coerulescens* green fluorescent protein (AcGFP)-tagged CD81 or SR-BI and the basal surface imaged by total internal reflection fluorescence (TIRF) microscopy (Fig. 2A and D). Flow cytometry confirmed comparable levels of CD81 and SR-BI expression in the transduced cells compared to endogenous protein in primary human hepatocytes (PHH) and Huh-7.5 hepatoma cells (mean fluorescent intensity of CD81 on PHH – 103, HepG2-CD81 – 94, Huh-7.5 – 99 and SR-BI on PHH – 89, HepG2-CD81 – 99, Huh-7.5 – 104). In polarized HepG2 cells, the mobile fraction of CD81 was significantly reduced (polarized *Mf* = 52% versus non-polarized *Mf* = 67%). Furthermore, the median CD81 diffusion coefficient was similarly reduced in polarized cells (polarized DC = 0.07 μm^2^ s^−1^ versus non-polarized DC *=* 0.11 μm^2^ s^−1^) (Fig. 2C). In contrast, SR-BI FRAP values were comparable in polarized and non-polarized HepG2 cells (Fig. 2E and F). Comparable DC values for AcGFP-tagged CD81 and SR-BI were observed in non-polarized PHH and two independent hepatoma cell lines, Huh-6 and Huh-7.5 (Table 1).

**Table 1:** Diffusion rates of CD81, SR-BI and HCVpp in different cells assessed by FRAP and SPT

Methodology	Cell	Track	Diffusion coefficient (DC), μm^2^ s^−1^						Significance^b^

			Polarized			Non-polarized			
	
			Median	IQR	*n*^a^	Median	IQR	*n*	
FRAP	HepG2	CD81	0.07	0.13	250	0.11	0.24	250	***
		SR-BI	0.16	0.24	250	0.14	0.36	250	
	Huh-7.5	CD81	–	–	–	0.09	0.15	250	
		SR-BI	–	–	–	0.13	0.58	250	
	Huh-6	CD81	–	–	–	0.12	0.29	250	
		SR-BI	–	–	–	0.15	0.29	250	
	PHH	CD81	–	–	–	0.10	0.21	250	
		SR-BI	–	–	–	0.14	0.48	250	
SPT	HepG2	CD81	0.11	0.11	3000	0.17	0.16	3000	***
		CD81_ΔC_	0.10	0.13	2000	0.10	0.13	2000	
		HCVpp on HepG2.CD81	0.07	0.21	1500	0.14	0.25	1500	***
		HCVpp on HepG2.CD81_ΔC_	0.05	0.14	1500	0.09	0.30	1500	

a *n*, number of events measured.

b Comparison of median DC in polarized and non-polarized cells (Mann–Whitney test, ***P ≤ 0.001).

**Fig. 3:**
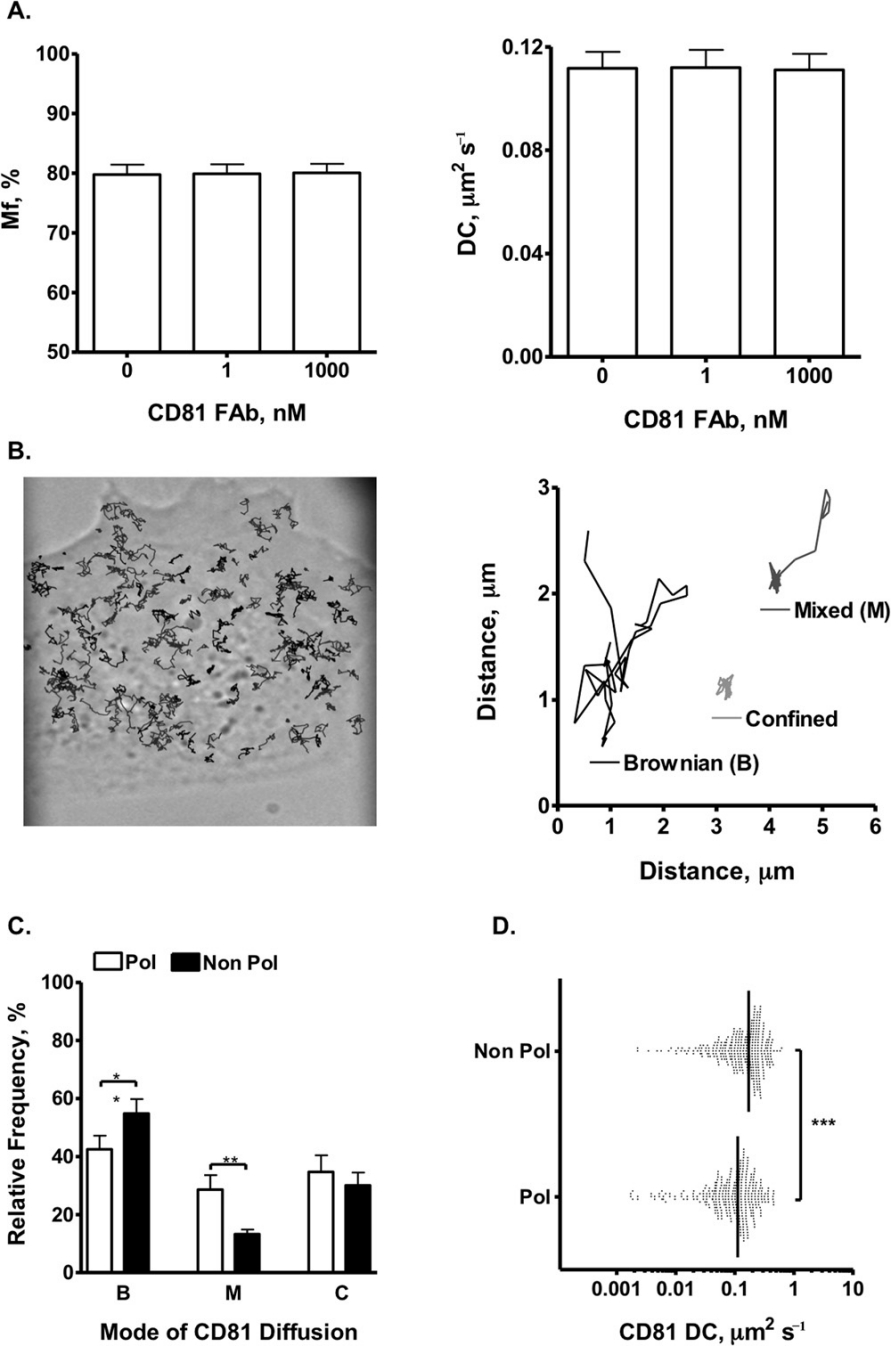
Single particle tracking of CD81 in polarized and non-polarized HepG2 cells. Initial studies assessed the effect of anti-CD81 2s66 Fab on AcGFP–CD81 mobility by FRAP methodology. Anti-CD81 2s66 Fab was pre-incubated with cells for 1 h prior to FRAP imaging. The Fab had no significant effect on CD81 mobility or immobile fraction of DC (A). The image (B) shows CD81 trajectories at the non-polarized HepG2 cell surface and the cartoon depicts representative trajectories with Brownian (B), mixed (M) or confined (C) behaviour. The relative frequency of anti-CD81 2s66 trajectories in polarized (Pol – white bars) and non-polarized (Non Pol – black bars) cells is shown (C). Scatter plots for diffusion coefficients calculated from the MSD-τ plots for Brownian trajectories in polarized and non-polarized cells, where each point represents one trajectory with the black line indicating the median, are represented (D). Total and Brownian CD81 DCs are summarized in Table 1. BMC sampling was utilized to present equivalent trajectories per parameter, where a minimum of 10 cells and 250 trajectories were measured. Non-parametric Mann–Whitney *t*-tests were used to determine the degree of significance (*P < 0.05, **P < 0.001, ***P < 0.0001).

To expand and verify the population-based FRAP experiments that do not account for stochastic events such as flow or constraints within the membrane, we measured CD81 dynamics by SPT anti-CD81 Fabs labelled with a single Atto647N fluorescent probe, as previously reported for tetraspanin CD9 (Espenel *et al*., 2008). We selected two anti-CD81 mAbs 2s66 and 2s155 specific for linear (amino acids 173–192) and conformation-dependent epitopes, respectively, in the large extracellular loop. We confirmed that both labelled Fabs bound CD81 and neither Fab affected CD81 mobility or DC by FRAP at concentrations up to 1 μg ml^−1^ (Fig. 3A). Live TIRF microscopic imaging of cell surface bound anti-CD81 Fab fragments enabled us to quantify CD81 dynamics at the basal membrane with high temporal (100 ms) and spatial (lateral, 50 nm) resolution. Figure 3B represents the distribution of individual CD81 trajectories at the non-polarized HepG2 cell membrane (50–100 trajectories per cell) calculated using a linear fit to the mean squared displacement (MSD) versus time plots. The mode of diffusion was evaluated within a trajectory using an algorithm based on a neural network trained to detect Brownian, confined and directed trajectories. Each identified segment was analysed using MSD versus time plots (for details, see *Experimental procedures*, and for an example, see Fig. S1). Three modes of diffusion were observed for CD81 trajectories (Fig. 3C): (i) Brownian diffusion (55 ± 5% of total trajectories) with a median DC value of 0.17 μm^2^ s^−1^, (ii) confined or restricted diffusion (30 ± 5% of total trajectories) with a low median DC value of 0.03 μm^2^ s^−1^ and a confinement diameter of 367 nm on average and (iii) diffusion with different combinations of Brownian and confined modes referred to as ‘mixed trajectories’ (15 ± 2% of total trajectories). Under our experimental conditions, CD81 did not show any directed diffusion. Comparable data were observed with both anti-CD81 Fabs (Fig. S2). Approximately 30% of confined trajectories overlapped with ensemble anti-CD81 and anti-CD9 staining of non-polarized HepG2 cells, demonstrating that sites of single particle confinement overlap with areas of enriched tetraspanin staining or TEMs as previously reported for CD9 (Espenel *et al*., 2008). HepG2 polarization reduced the proportion of CD81 trajectories showing Brownian behaviour, while increasing the frequency of molecules with mixed diffusion (Fig. 3C). Furthermore, we observed a significant reduction in the diffusion coefficient of Brownian diffusing CD81 molecules in polarized HepG2 compared to non-polarized cells (Fig. 3D, Table 1). In summary, these studies show that polarization slows CD81 diffusion and increases protein confinement.

### HepG2 polarization limits HCVpp dynamics

**Fig. 4:**
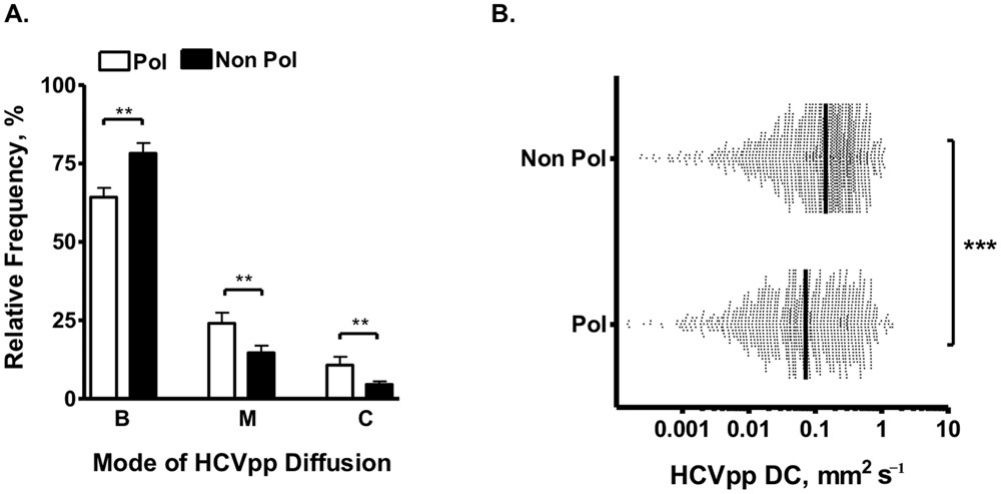
Single particle tracking of HCVpp in polarized and non-polarized HepG2 cells. Relative modes of Brownian (B), mixed (M) and confined (C) HCVpp diffusion at the polarized (Pol – white bars) and non-polarized (Non Pol – black bars) HepG2 cell membrane (A). A minimum of 10 cells and 250 trajectories were measured per parameter as predetermined by BMC sampling. Scatter plots for HCVpp diffusion coefficients bound to polarized or non-polarized HepG2 cells were calculated from the MSD-τ plots for all Brownian trajectories, where each point represents one trajectory with the black line indicating the median (B). Total and Brownian HCVpp DCs are summarized in Table 1. Non-parametric Mann–Whitney *t*-tests were used to determine the degree of significance (*P < 0.05, **P < 0.001, ***P < 0.0001).

To investigate whether HepG2 polarization limits HCV diffusion, we used lentiviral pseudotypes expressing HCV-encoded E1E2 glycoproteins to study particle trafficking. Lentiviral cores were identified by their incorporation of GFP-Vpr (Campbell *et al*., 2007) and HCV E1E2 containing particles by their reactivity with anti-HCV E2 11/20 Fab-Atto647N. Anti-HCV E2 11/20 only bound HCVpp showing no reactivity with envelope deficient or VSV-G expressing lentivirus particles (Fig. S3). Of GFP-Vpr-positive particles, 79% bound anti-HCV E2, showing that the majority of HCVpp incorporate HCV glycoproteins. Given the limited lifetime of the GFP fluorescent signal we were only able to track particle diffusion with the Atto-labelled anti-HCV E2 Fab. We confirmed that the concentration of anti-HCV E2 Fab (5 pM) required to track particles had no effect on HCVpp infectivity. We noted that in non-polarized HepG2 cells, the dominant mode of HCVpp diffusion within the first hour post inoculation was Brownian (78 ± 3%), with mixed (15 ± 2%) and confined (5 ± 1%) trajectories comprising a minor component of the population (Fig. 4A). HepG2 polarization led to an increase in mixed and confined trajectories and a concomitant decrease in the frequency of HCVpp trajectories with Brownian diffusion (Fig. 4A, Table 1). Furthermore, HCVpp Brownian DC was significantly reduced on polarized HepG2 cells compared to non-polarized cells, with speeds of 0.14 μm^2^ s^−1^ and 0.07 μm^2^ s^−1^, respectively (Fig. 4B, Table 1), highlighting the similar effect(s) of HepG2 polarization on HCVpp and CD81 dynamics.

### The CD81 C-terminus regulates protein and HCVpp dynamics

**Fig. 5:**
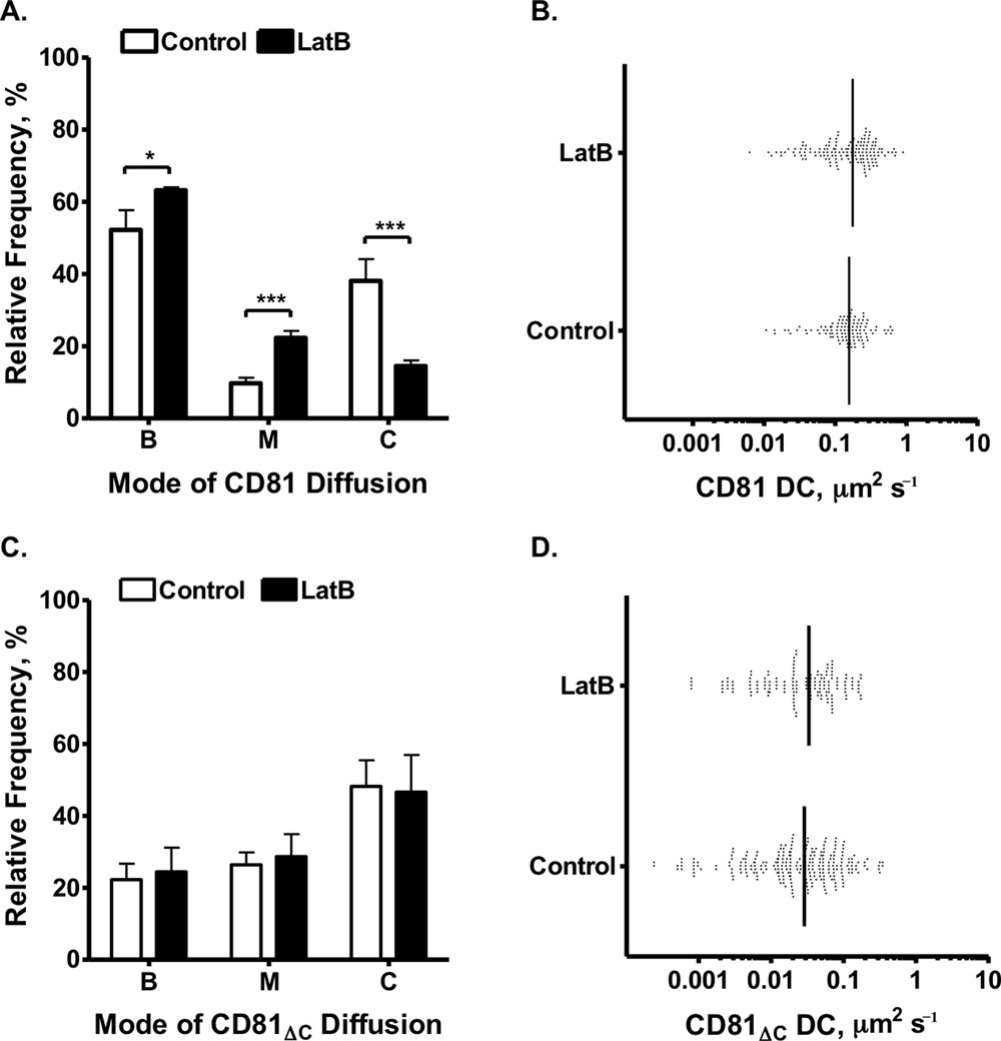
The role of F-actin in CD81 mobility. The relative frequency of anti-CD81 2s66 trajectories in LatB (1 μM) (black bars) or DMSO control (white bars) treated non-polarized HepG2 cells expressing wild-type (A) or CD81_ΔC_ (C). Scatter plots for wild-type (B) or CD81_ΔC_ (D) diffusion coefficients calculated from the MSD-τ plots for Brownian trajectories in treated or untreated cells are shown, where each point represents one trajectory with the black line indicating the median. Non-parametric Mann–Whitney *t*-tests were used to determine the degree of significance (*P < 0.05, **P < 0.001, ***P < 0.0001).

Given the many reports that membrane protein association with filamentous actin (F-actin) limits their mobility (Kusumi *et al*., 1993; Sako *et al*., 1998; Treanor *et al*., 2011) and cell polarization is accompanied by changes in the underlying actin cytoskeleton (Delorme-Axford and Coyne, 2011), we were interested to determine a role for actin in CD81 dynamics. The tight junction complex between polarized epithelia is closely associated with the actin cytoskeleton and subtle changes to the cortical actin cytoskeleton can lead to a loss of junctional integrity and barrier formation. Inhibition of actin polymerization with latrunculin B (LatB) reduced HepG2 tight junction integrity (data not shown) and we were only able to study the effect of LatB on CD81 dynamics in non-polarized HepG2 cells. LatB decreased the frequency of confined trajectories with an associated increase in the frequency of mixed and Brownian diffusing trajectories (Fig. 5A). LatB had no effect on CD81 Brownian DC (Fig. 5B), consistent with a role for actin polymers to confine CD81 mobility.

**Fig. 6:**
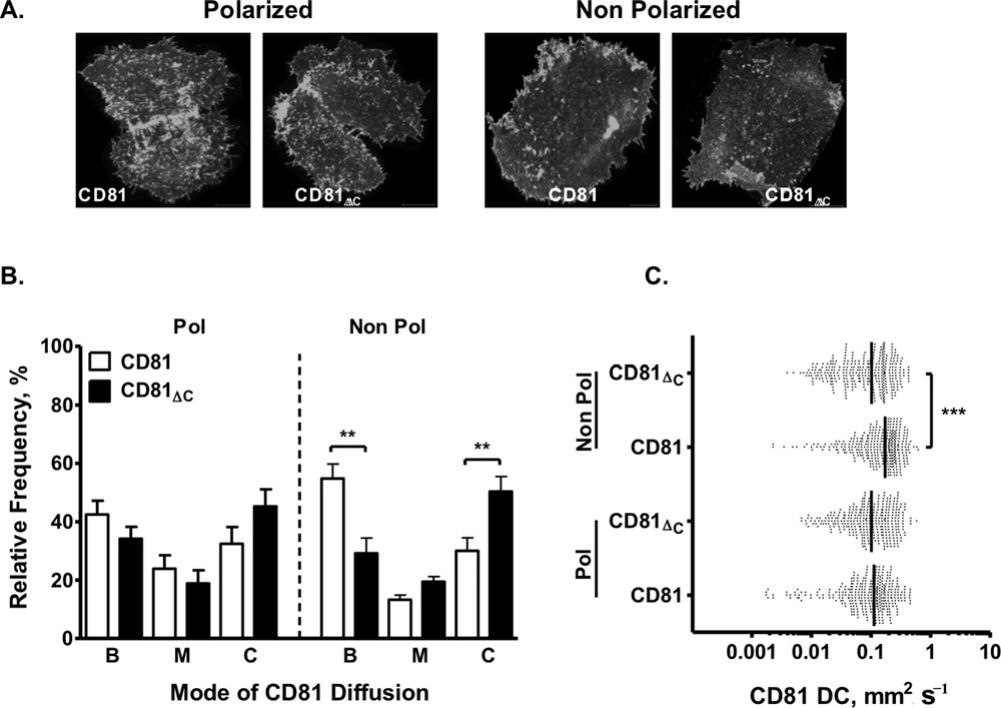
CD81 C-terminus regulates protein diffusion and confinement. HepG2 cells were transfected to express wild-type or CD81_ΔC_, and representative confocal images of polarized (day 7 post plating with a polarization index of 44%) and non-polarized (day 1 post plating with a polarization index of 1%) HepG2 cells taken at the cell–glass interface are shown (A). The images were acquired using a 100× 1.4NA objective on the Zeiss LSM 780 confocal with 0.8 μm Z sections. The relative frequency of wild-type (white bars) and CD81_ΔC_ (black bars) Brownian (B), mixed (M) or confined (C) trajectories on polarized (B: 42 ± 5%, 34 ± 4%, M: 24 ± 5%, 19 ± 4%, C: 32 ± 6%, 45 ± 6%) and non-polarized (B: 55 ± 5%, 29 ± 5%, M: 13 ± 2%, 20 ± 2%, C: 30 ± 5%, 50 ± 5%) HepG2 cells is shown (B). Scatter plots of CD81 and CD81_ΔC_ Brownian DC calculated from the MSD-τ plots for Brownian trajectories in polarized (0.11, 0.10 μm^2^ s^−1^) and non-polarized (0.17, 0.10 μm^2^ s^−1^) cells are shown, where each point represents one trajectory with the vertical line indicating the median (C). Total and Brownian CD81 DCs are summarized in Table 1. A minimum of 10 cells and 250 trajectories were measured per parameter as determined by BMC sampling. Non-parametric Mann–Whitney tests were used to determine the degree of significance (*P < 0.05, **P < 0.001, ***P < 0.0001).

**Fig. 7:**
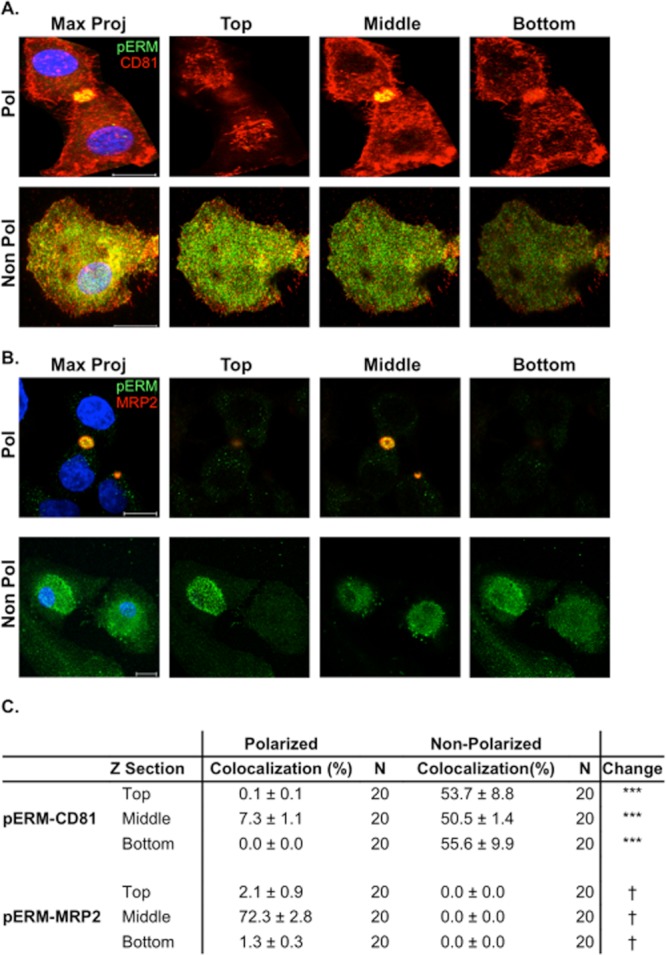
Phosphorylated ERM and CD81 localization in polarized and non-polarized HepG2 cells. Confocal images of HepG2 cells costained with anti-phosphorylated ERM (pERM) (green) and anti-CD81 (red) (A) or anti-MRP2 (red) (B). Sixteen bit images were acquired with optimal pixel resolution using a 100× Plan Apochromat 1.4NA oil immersion objective on a Zeiss LSM 780 confocal microscope. Representative maximum projections, constructed from Z sections (0.44 μm thick sections per cell) of polarized and non-polarized HepG2 cells, are shown. Representative Z sections from the top, middle or bottom of the cell are shown, with the bottom being closest to the glass. Cell nuclei were counter-stained with DAPI. Scale bars represent 10 μm. Quantitative pERM–CD81 and pERM–MRP2 colocalization analysis at different Z positions (top, middle and bottom) were calculated in accordance with Zinchuck *et al*. The colocalization coefficient is weighted for either CD81 or MRP2 expression (C), with the background threshold determined by the fluorescent signal of control isotype-matched IgG-stained HepG2 cells and set as twice the background fluorescent signal plus 3 SD. The change in colocalization between polarized and non-polarized cells was compared using a Mann–Whitney test (***P < 0.001); no MRP2 expression was detected in non-polarized cells (†).

The CD81 C-terminal region has been reported to interact with the actin cytoskeleton via ERM (Sala-Valdes *et al*., 2006; Chang and Finnemann, 2007; Coffey *et al*., 2009). To investigate the role of the CD81 C-terminus in regulating protein mobility we expressed a mutant protein lacking the C-terminal region (CD81_ΔC_) in HepG2 cells and confirmed that LatB had no effect on its dynamics (Fig. 5C and D). We noted a significant reduction in the frequency of Brownian CD81_ΔC_ trajectories compared to wild-type protein (Fig. 5C), prompting us to study mutant protein localization, dynamics and viral receptor activity. Wild-type and mutant CD81 showed a comparable staining pattern in polarized and non-polarized HepG2 cells (Fig. 6A). We confirmed that CD81_ΔC_ showed an increased frequency of confined trajectories and a loss of Brownian diffusing molecules compared to wild-type protein in non-polarized cells (Fig. 6B). In addition, Brownian CD81_ΔC_ trajectories diffused at a significantly slower speed than wild-type protein (Fig. 6B and C, Table 1). In contrast, we failed to observe any significant differences between wild-type and CD81_ΔC_ dynamics in polarized HepG2 (Fig. 6, Table 1). We confirmed that neither protein affected the ability of HepG2 cells to polarize or to retain 5-chloromethylfluorescein diacetate (CMFDA) at apical lumens, a marker of tight junction integrity. These observations support two roles for the CD81 C-terminus in non-polarized cells: (i) to limit CD81 diffusion in an actin-dependent manner (Fig. 5) and (ii) to promote Brownian CD81 diffusion (Fig. 6C) via a potentially actin-independent mechanism. The ERM family of actin binding proteins plays an important role in stabilizing apical microvilli and has been reported to localize to the apical domains of polarized airway cells (Berryman *et al*., 1993; Berbari *et al*., 2009). We therefore studied the localization of phosphorylated ERM in polarized and non-polarized HepG2 cells. We noted a significant reorganization of phosphorylated ERM to the MRP2-positive apical membrane in polarized HepG2 and a reduction in CD81–ERM colocalization at the basal membrane (Fig. 7).

**Fig. 8:**
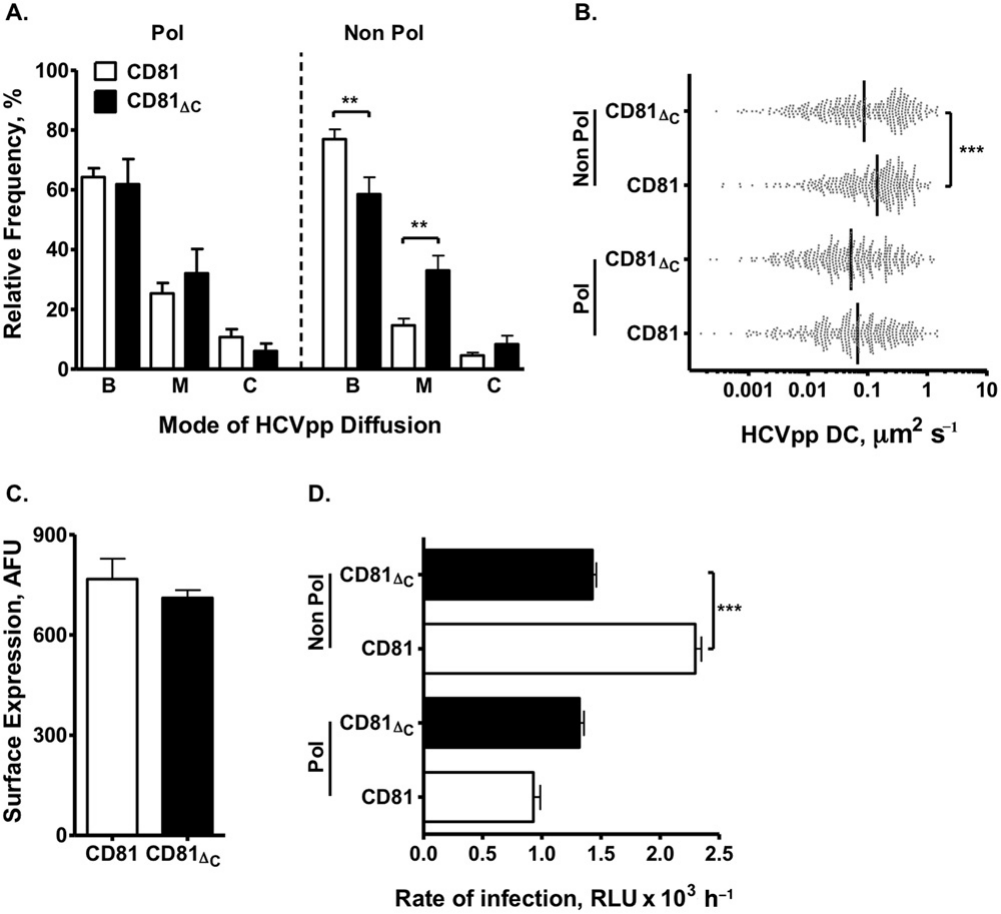
CD81 C-terminal tail regulates HCVpp dynamics and HCV infection. The relative frequency of Brownian (B), mixed (M) or confined (C) HCVpp trajectories on polarized and non-polarized HepG2 cells expressing wild-type (white) and CD81_ΔC_ (black) protein (A). Scatter plots for HCVpp diffusion coefficient (DC) in polarized and non-polarized HepG2 cells expressing wild-type or CD81_ΔC_ are calculated from the MSD-τ plots for Brownian trajectories (B). Each point represents one trajectory with the vertical line indicating the median. A minimum of 10 cells and 250 trajectories were measured per parameter as predetermined by BMC sampling. Non-parametric Mann–Whitney *t*-tests were used to determine the degree of significance (*P < 0.05, **P < 0.001, ***P < 0.0001). Total and Brownian CD81 DCs are summarized in Table 1. Cell surface expression of wild-type and CD81_ΔC_ in non-polarized HepG2 cells (C). Fifty cells were imaged under the same conditions and the data expressed as arbitrary fluorescence units (AFU). The rate of HCVpp infection of polarized and non-polarized HepG2 cells expressing wild-type and CD81_ΔC_, where the data are expressed as luciferase relative light units per hour (D). The data are representative of three independent experiments where infections were performed in triplicate. A one-way anova with a Tukey post test was used to determine the degree of significance (***P < 0.0001).

To ascertain whether the reduced HCVpp dynamics observed at the basolateral membrane is CD81-dependent we compared HCVpp mobility on HepG2 cells expressing wild-type and CD81_ΔC_ proteins. We observed a significant reduction in the proportion of Brownian diffusing HCVpp and an increased number of particles with mixed or confined diffusion on non-polarized HepG2 expressing CD81_ΔC_ compared to wild-type protein (Fig. 8A, Table 1). Furthermore, the Brownian diffusion coefficient of HCVpp on non-polarized HepG2 cells expressing CD81_ΔC_ was significantly reduced compared to cells expressing wild-type CD81 (Fig. 8B). In contrast, HCVpp diffusion modes and speed were comparable on polarized HepG2 expressing wild-type or CD81_ΔC_ (Fig. 8A and B). To ascertain whether the altered pseudoparticle dynamics observed on non-polarized HepG2–CD81_ΔC_ impacts virus infection, we compared infection of HepG2 cells expressing wild-type or CD81_ΔC_ proteins. We confirmed that wild-type and mutant proteins were expressed at comparable levels (Fig. 8C). HCVpp infected non-polarized cells expressing CD81_ΔC_ with significantly reduced efficiency compared to cells expressing wild-type protein. In contrast, polarized HepG2 expressing wild-type or mutant CD81 supported comparable rates of infection (Fig. 8D). In summary, these data support a role for CD81 dynamics in regulating HCV entry into hepatoma cells that is dependent on their polarized status.

## Discussion

Our live cell imaging studies highlight the dynamic nature of CD81 and SR-BI as reported for tetraspanin CD9 (Espenel *et al*., 2008). The principal new observations from these studies are that hepatoma polarization restricts CD81, lipid and HCVpp mobility, providing a mechanism to explain the limited HCV infection of polarized cells (Mee *et al*., 2009). Our FRAP studies show similar CD81 diffusion coefficient values to those measured by SPT and confirm that HepG2 polarization limits CD81 and DiD/DiO lipid dynamics. In contrast, SR-BI mobility was not altered by HepG2 polarization, possibly reflecting the biological function of SR-BI to remove cholesterol esters from lipoproteins into the local plasma membrane that may overcome global changes in membrane fluidity (Acton *et al*., 1996; Swarnakar *et al*., 1999).

Our studies demonstrate a role for the CD81 C-terminal region to regulate protein dynamics that is dependent on the polarized status of the cell, possibly reflecting altered cholesterol and/or lipid constituents of the cell membrane (Zegers and Hoekstra, 1998). Our observation that CD81_ΔC_ has reduced viral receptor activity in non-polarized HepG2 cells is consistent with an earlier report by Bertaux and colleagues demonstrating reduced HCVpp infection of Huh-7 cells (Bertaux and Dragic, 2006). The observation that CD81_ΔC_ diffuses slower than wild-type and supports reduced HCVpp infection of non-polarized HepG2 cells suggests that CD81 lateral diffusion speed may limit HCV internalization. In addition, the mutant CD81 protein may show altered interaction(s) with accessory proteins such as claudin-1 or epidermal growth factor receptor that play a role in HCV infection (Lupberger *et al*., 2011; L. Zona *et al*., submitted). The reduced diffusion of HCVpp observed on HepG2 cells expressing CD81_ΔC_ suggests a role for CD81 in regulating pseudoparticle diffusion at the non-polarized cell membrane. Finally, the ability of HCVpp to infect HepG2 expressing CD81_ΔC_ with comparable efficiency independent of their polarized status highlights a potential role for this domain in restricting HCV infection of polarized cells, in contrast to the many reports suggesting that infection is limited by the access of virus to tight junction proteins claudin-1 and occludin (Brazzoli *et al*., 2008; Burlone and Budkowska, 2009; Delorme-Axford and Coyne, 2011).

Tetraspanin proteins have received significant attention as gateways for the assembly and entry of various pathogens: including HIV-1, influenza and the malarial parasite plasmodium, providing potential targets for therapeutic intervention (Monk and Partridge, 2011). Our observation that HepG2 polarization reduces CD81 mobility and increases the frequency of confined trajectories may have implications for other pathogens that utilize TEMs to enter or exit their host cell and highlight the importance of studying membrane protein dynamics in polarized cells.

It is noteworthy that the diffusion speed of HCVpp closely parallels the CD81 diffusion coefficient on non-polarized cells (Table 1). Although HCVpp diffusion values 0.07–0.14 μm^2^ s^−1^ are comparable to those reported for the related Dengue virus (van der Schaar *et al*., 2008), they are significantly faster than other characterized viruses, e.g. human papilloma virus type 16 and murine polyoma virus (Ewers *et al*., 2005; Schelhaas *et al*., 2008; Burckhardt and Greber, 2009; Burckhardt *et al*., 2011), which may reflect the different dynamics of specific viral receptors. Several viruses have been reported to use actin retrograde flow to infect host cells (reviewed in Burckhardt *et al*., 2011). Coller *et al*. reported the localization of fluorescent lipid DiD-labelled HCV particles with CD81, suggesting directed particle diffusion along filopodia in Huh-7 hepatoma cells (Coller *et al*., 2009). In contrast, our SPT studies detected a predominantly Brownian mode of HCVpp and CD81 diffusion, with no directed actin-dependent trajectories observed. The differences between these studies may reflect distinct cell types, structures being studied or imaging techniques utilized. However, it is noteworthy that the CD81 diffusion modes we observed are consistent with reports of other plasma membrane proteins (Suzuki *et al*., 2007; Sergé *et al*., 2008). The negligible frequency of directed CD81 trajectories may reflect protein internalization (Farquhar *et al*., 2012). Espenel *et al*. reported a similar lack of directed CD9 diffusion in PC3 human prostate and HeLa cancer cells, suggesting that this may be a common property of tetraspanin proteins (Espenel *et al*., 2008).

The functional importance of the CD81 second large extracellular loop in binding HCV is well recognized (Zhang *et al*., 2004; Bertaux and Dragic, 2006); however, the C-terminal tail has received less attention. The CD81 C-terminus contains 12 amino acids that are conserved across several animal species, suggesting functional importance; however, no signalling motifs have been demonstrated in this short cytoplasmic region (Hemler, 2005). A recent report by Wang and colleagues highlighted an essential role for the CD9 C-terminal tail in tetraspanin function and molecular organization (Wang *et al*., 2011). Hence, our results showing a role for the C-terminus in CD81 mobility may be widely applicable to other tetraspanin proteins; for example, the C-terminal tail of CD151 was reported to support integrin-dependent adhesion strengthening and cell morphology (Lammerding *et al*., 2003).

Our data showing that LatB inhibition of actin polymerization decreased the frequency of confined CD81 trajectories is consistent with the literature describing a tethering function for F-actin to confine membrane proteins. Furthermore, the negligible effect of LatB on CD81_ΔC_ dynamics confirms a role for the C-terminus in linking CD81 to the actin cytoskeleton (Sala-Valdes *et al*., 2006). However, SPT experiments showing reduced CD81_ΔC_ DC in non-polarized cells highlight an additional role for this region in promoting protein diffusion. Our observation that phosphorylated ERM proteins localize predominantly to the apical lumen of polarized HepG2 cells is consistent with reports in polarized airway cells (Berryman *et al*., 1993; Berbari *et al*., 2009), consistent with the reduced colocalization of CD81–pERM observed in polarized cells. A recent study by Orlando and colleagues reporting a role for the actin cytoskeleton to both promote and restrict adhesion molecule L1CAM dynamics may provide a model for CD81 C-terminal–cytoskeletal interactions. The authors demonstrated a dual role for the L1CAM C-terminal tail interacting with ERM or cytoskeletal adaptor protein ankyrin, where ERM interaction promoted actin-dependent retrograde L1CAM movement, whereas ankyrin confined protein movement (Sakurai *et al*., 2008; Brachet *et al*., 2010). Thus, local concentrations of ERM at the basal membrane may provide a switch to regulate CD81 C-terminal tail interactions with other components of the cytoskeleton.

In summary, our data suggest that CD81 lateral diffusion may limit HCV entry. We propose that CD81 needs to freely diffuse in the cellular membrane to form transient associations with claudin-1 (Harris *et al*., 2008; 2010). Our recent observation that HCV promotes CD81 and claudin-1 co-endocytosis supports a model where virus–CD81 complexes move laterally across the plasma membrane prior to internalizing (Farquhar *et al*., 2012). Cao and colleagues reported that hepatocytes sequester or pre-recruit structural and enzymatic components of the clathrin-based endocytic machinery to functional hot spots on the basal membrane (Cao *et al*., 2011). These sites of endocytic uptake may overlap with areas of CD81 confinement and particle internalization at the basal membrane. Our observation that polarization reduces the dynamics of both CD81 and HCVpp at the basolateral membrane uncovers a new pathway for polarized epithelia to restrict pathogen invasion and highlights the importance of studying virus–host cell interactions in polarized cell systems.

## Experimental procedures

### Cell lines and reagents

HepG2 cells were propagated in Dulbecco's modified Eagle medium (DMEM) supplemented with 10% fetal bovine serum and 1% non-essential amino acids. HepG2 cells naturally lack CD81 expression and were transduced to express exogenous human CD81, CD81 lacking a C-terminal region (CD81_ΔC_) or AcGFP-tagged CD81 or SR-BI as previously described (Harris *et al*., 2008). Latrunculin B (LatB) was purchased from Sigma and fluorescent membrane dyes DiO-C18 and DiD-C18 from Invitrogen. Fab fragments of anti-CD81 monoclonal antibodies 2s66, 2s155 (Harris *et al*., 2008), anti-HCV E2 11/20 (Hsu *et al*., 2003) were prepared using papain cleavage and labelled with single photostable fluorophores, Atto647N or Cy3B, purchased from Atto-Tech or GE HealthCare, as previously described (Espenel *et al*., 2008).

### Quantification of HepG2 polarity

HepG2 cells were grown on 13 mm diameter borosilicate glass coverslips (Fisher Scientific, UK) to the time point required and fixed in 3% paraformaldehyde at room temperature for 30 min. Cells were stained for apical multidrug-resistant protein 2 (MRP2) (anti-MRPR clone M2 III-6; Abcam Cambridge, UK) in 0.1% Triton (Sigma-Aldrich, UK), 0.5% bovine serum albumin (BSA) in phosphate-buffered saline (PBS) and Alexa-Fluor 488-conjugated goat anti-mouse. Cell nuclei were visualized using 4′, 6′-diamidino-2-phenylindole (DAPI; Invitrogen). The polarity index was determined by counting the number of MRP2-positive apical structures per 100 nuclei using a Nikon Eclipse TE2000-S fluorescence microscope as previously reported (Mee *et al*., 2009). Polarity indices of 40–45 are routinely observed 7 days post plating. Since at least two cells are required to form a bile canaliculus, this corresponds to at least 80–90% of cells developing polarity.

### Hepatitis C virus pseudoparticle genesis and infection

Pseudoviruses expressing luciferase or GFP-Vpr were generated as previously reported (Hsu *et al*., 2003; Campbell *et al*., 2007). Briefly, 293T cells were transfected with an equal ratio of plasmids encoding HIV provirus expressing luciferase, GFP-Vpr and HCV strain H77 E1E2 envelope glycoproteins, VSV G or empty vector. Supernatants were harvested 48 h post transfection, pooled and filtered. Virus containing media was added to target cells plated at 1.5 × 10^4^ cells cm^−2^ for 8 h, unbound virus was removed by washing every 30 min and the cells incubated at 37°C. Seventy-two hours post infection the cells were lysed, substrate added and luciferase activity measured for 10 s in a luminometer (Lumat LB 9507). Specific infectivity was calculated by subtracting the mean Env-pp relative light unit (RLU) signal from the HCVpp signal.

### Fluorescence recovery after photobleaching (FRAP)

Cells transduced to express AcGFP.CD81 and AcGFP.SR-BI were plated onto Mattek glass bottomed dishes at a density of 1.5 × 10^4^ cells cm^−2^ and imaged using a 100× Plan Apochromat 1.4NA oil immersion objective on a Zeiss LSM 780 confocal microscope with a GaAsP spectral detector. Sixteen bit images were attained with optimal pixel resolution. Tagged proteins were excited with the argon 488 laser (DiD-labelled membranes were excited with the 633 laser) and initial images acquired with low laser power (0.1–1% transmission), photobleaching was performed with full laser power for 20 iterations on selected circular regions of interest (ROIs) identified in the planar membrane. Subsequent recovery images were collected using low laser power at 0.18 s intervals for approximately 2 min, until ROI recovery had reached a plateau. Only a single Z section was bleached and imaged, typically the basal side of the cell contacting the coverslip. The mean fluorescent intensity over time was obtained for the photobleached ROI, a background ROI (containing no cells) and an unbleached ROI in the cell of interest. ROIs were selected using the Zeiss Zen analysis software. Changes in fluorescence following photobleaching were normalized for fluctuations in image capture, laser power and overall fluorescence loss in the cells by subtracting the background and unbleached ROI using the Zeiss Zen software. The values obtained for the photobleached ROI were converted to relative fractional recovery, where the prebleach fluorescence intensity values equal to 100%. Data were imported into GraphPad Prism and fitted using a single exponential decay algorithm, *Y* = span (1-exp (−K*X)) + plateau. The span and plateau were used to calculate mobile fraction. The diffusion coefficient (*D*) was calculated using a simple two-dimensional diffusion model for a circular bleach ROI: *D* = 0.224 × (radius2/t1/2) (Axelrod *et al*., 1976). Bootstrap Monte Carlo sampling demonstrated that a minimum of 10 cells and 100 ROIs were required to represent the population.

### Single particle tracking of anti-CD81 Fabs and pseudoparticle by total internal reflection fluorescence (TIRF) microscopy

A multicolour single-molecule imaging TIRF system, equipped with a Plan Fluor 100X/1,45 NA objective (Zeiss, Le Peck, France), was used for SPT studies. The total internal reflection angle was manually adjusted for each experiment. All experiments were performed with a 100 ms integration time. To achieve maximal specificity in the detection of two fluorescent signals, alternating-laser excitation was performed using an acousto-optical tuneable filter and controller (AOTF; AA Optoelectronics, Les Chevreuses, France) (Margeat *et al*., 2006). All movies were analysed using designated software, named ‘PaTrack’ implemented in visual C++. In our software, the detection, estimation of positions and reconnection of detected molecules are carried out sequentially. Briefly, the centre of each fluorescence peak was determined with sub-pixel resolution by fitting a two-dimensional elliptical Gaussian function with a background value. Trajectories are constructed in the sequence, frame after frame. The association of spots of sequential images within trajectories is based on a nearest-neighbour rule. Overlapping tracks are discarded or construction in the sequence of two fluorescent molecules is stopped when they overlap (to be kept and analysed, the length of the trajectories should be over 40 frames). However, the density of the particles per frame was sufficiently low to minimize overlapping. Apparent diffusion coefficient values were determined from a linear fit between the first and fourth points (D1–4) of the MSD versus time lag according to Kusumi *et al*. (1993). The mode of diffusion was determined using a new algorithm based on a back-propagation neural network that has been trained using synthetic trajectories to detect pure Brownian, confined and directed motion modes (H.J. Harris *et al*., manuscript in preparation). Thanks to a sliding window, the trajectory is analysed and the different modes can be confidently detected within a trajectory for segments larger than 10 frames. The algorithm has been tested with simulated trajectories displaying pure Brownian, confined or directed behaviour or a combination of these three modes and successfully applied to a set of single-molecule experiments previously recorded for tetraspanins (Espenel *et al*., 2008). For each identified segment, the MSD curve was linearly fitted (Brownian) or adjusted with a quadratic curve (4Dt + ν^2^t^2^) (directed diffusion) or exponential curve L2/3(1-exp(−12Dt/L2) (confined diffusion), where L is the side of a square domain, the confinement diameter being related to L by dconf = (2/√π)L).

### Single particle tracking CD81 diffusion

Cells were incubated with phenol red free supplemented DMEM at 37°C for 10 min with anti-CD81 Fab-Atto647N fragments (3 pM). Following incubation the cells were washed three times in phenol red free DMEM and single fluorophores visualized with the TIRF evanescent wave (∼ 100 nm depth) on the basal side of the cell contacting the coverslip. One thousand frame movies were attained with 100 ms integration time. Single fluorophores were identified by monitoring single-step fluorescence bleaching.

### Single particle tracking pseudoparticle diffusion

GFP.Vpr-labelled HCVpp were incubated with phenol red free supplemented DMEM at 37°C for 10 min with anti-HCV E2 11/20-Atto647N Fab fragments (5 pM) prior to cell inoculation. Cells were incubated with labelled virions for 10 min and unbound virus removed by washing prior to monitoring particle diffusion at the basal cell surface with the TIRF evanescent wave. One thousand frame movies were attained with 100 ms integration time. Virions expressing HCV glycoproteins were identified as those expressing GFP.Vpr and labelled with anti-HCV E2 11/20-Atto647N Fab (Fig. S3).

### Ensemble costaining

Cells were incubated in phenol red free DMEM at 37°C for 10 min with Cy3B-labelled anti-CD9/CD81 Fab fragments (3 nM) for ensemble labelling and Atto647N-labelled anti-CD81 Fab fragments (3 pM) for SPT experiments. A maximum projection of Cy3B from the 1000 frame movie provided the overall staining for CD9 or CD81 indicating domains of enriched expression. The ensemble labelling was overlayed with the SPT data and the frequency of colocalizing trajectories calculated.

### Protein colocalization

Quantitative colocalization was measured according to published work (Zinchuk *et al*., 2007). Sequential scanning confocal Z sections (∼ 0.44 μm thick) of cells were obtained with optimal pixel resolution using a 100× Plan Apochromat 1.4NA oil immersion objective on a Zeiss LSM 780 confocal microscope. Microscope settings were optimized for each fluorescent protein to obtain the highest signal to noise ratio, while controlling for cross-talk. The background fluorescent signal was determined by measuring the signal of control isotype-matched IgG binding to cells. The background threshold was set at twice the background fluorescent signal plus 3 standard deviations. The cells being analysed were extracted from the whole image and single Z sections analysed using the colocalization tool in the Zeiss Zen software. Since the proteins are not expressed equally throughout the cell, the Mander's overlap coefficient and colocalization coefficient m1 and m2, respectively (Zinchuk *et al*., 2005), were utilized instead of the Pearson's correlation coefficient. The results are displayed as the weighted colocalization of CD81 or MRP2.

### Cell surface expression of proteins

Confocal Z sections (0.44 μm) of cells labelled with saturating levels of anti-CD81 Fab-Atto647N (40 μM) and membrane dye DiO (1 μg ml^−1^) were acquired with optimal pixel resolution using a 100× Plan Apochromat 1.4NA oil immersion objective on a Zeiss LSM 780 confocal microscope. The plasma membrane expression of CD81 was quantified using the overlay and intensity tools within the Zeiss Zen software. DiO staining was used to identify the plasma membrane and the fluorescence of bound anti-CD81 Fab defined protein expression level as arbitrary fluorescence units (AFU).

### Statistics – bootstrap Monte Carlo (BMC) sampling

The mobile fraction and diffusion coefficient of CD81 calculated from FRAP and SPT studies in non-polarized HepG2 cells were randomized and 10 samples with increasing number of values (*n*) collected. The coefficient of variation was calculated for the 10 samples with increasing *n*, demonstrating that 10 samples with a minimum of 250 trajectories for single-molecule studies and 100 ROI from 10 cells for FRAP studies were sufficient to represent the population. The correct choice of equation was calculated by running Akaikes information criterion and extra sum of squares *F*-test, assuming simpler model/equation unless a *P*-value less than 0.05. Given the non-Gaussian distribution of CD81 and HCVpp SPT dynamics we compared the median and interquartile range values between cells. The infection data are represented as the mean ± SEM unless otherwise stated, as the values fit a Gaussian distribution. Corrections for multiple sampling (Bonferroni method and Dunnett's multiple comparison test) were used where appropriate. Statistical analyses were carried out in the Prism 5 package (GraphPad software, San Diego, CA), and probabilities are represented as *P* < 0.05 (*), *P* < 0.01 (**) and *P* < 0.001 (***).

## Supporting information

**Fig. S1.** MSD plot and movie, movie of CD81 dynamics and MSD analysis. Example of CD81 single-molecule tracking using Atto647N-labelled Fab fragments (A). A neural network (see *Experimental procedures*) is used to determine CD81 behaviour within a trajectory. Each identified segment is then analysed using MSD analysis and a plot of Brownian (B) and confined (C) segments. The first four points are used to determine the apparent diffusion coefficient of the molecule. The fit of the confined trajectory has been performed using 50% of the dots.

**Fig. S2.** CD81 single-molecule tracking profiles. The relative frequency of CD81 trajectories with Brownian (B), mixed (M) or confined (C) behaviour observed with anti-CD81 Fabs 2s66 and 2s155 (A). A minimum of 10 cells and 250 trajectories were measured per parameter as predetermined by BMC sampling. Scatter plots for Brownian CD81 diffusion coefficients measured with anti-CD81 Fabs 2s66 or 2s155 in non-polarized HepG2 cells, where the median Brownian DC values for 2s66 and 2s155 are 0.17 ± 0.2 μm^2^ s^−1^ and 0.16 ± 0.2 μm^2^ s^−1^ respectively (B). Each point represents one trajectory with the vertical line indicating the median.

**Fig. S3.** Anti-HCV E2 specificity. Representative TIRF images of GFP.VPR HCVpp, Env-pp or VSV Gpp bound to non-polarized HepG2 cells costained with anti-HCV E2 11/20 Fab-Atto647N. Seventy-nine per cent of GFP.VPR expressing HCVpp costained with anti-HCV E2 11/20. The threshold for the green and red signals was defined as twice the mean background plus 3 standard deviations of uninfected HepG2 cells. Colocalization was established following the analysis of 40 cells. Scale bar represents 10 μm.
