# Supplementary figures and images for: Hepatoma polarization limits CD81 and hepatitis C virus dynamics
Source: Cell Microbiol. 2012 Nov 20;15(3):430–45. doi: 10.1111/cmi.12047 (PMC3599488; doi:10.1111/cmi.12047)

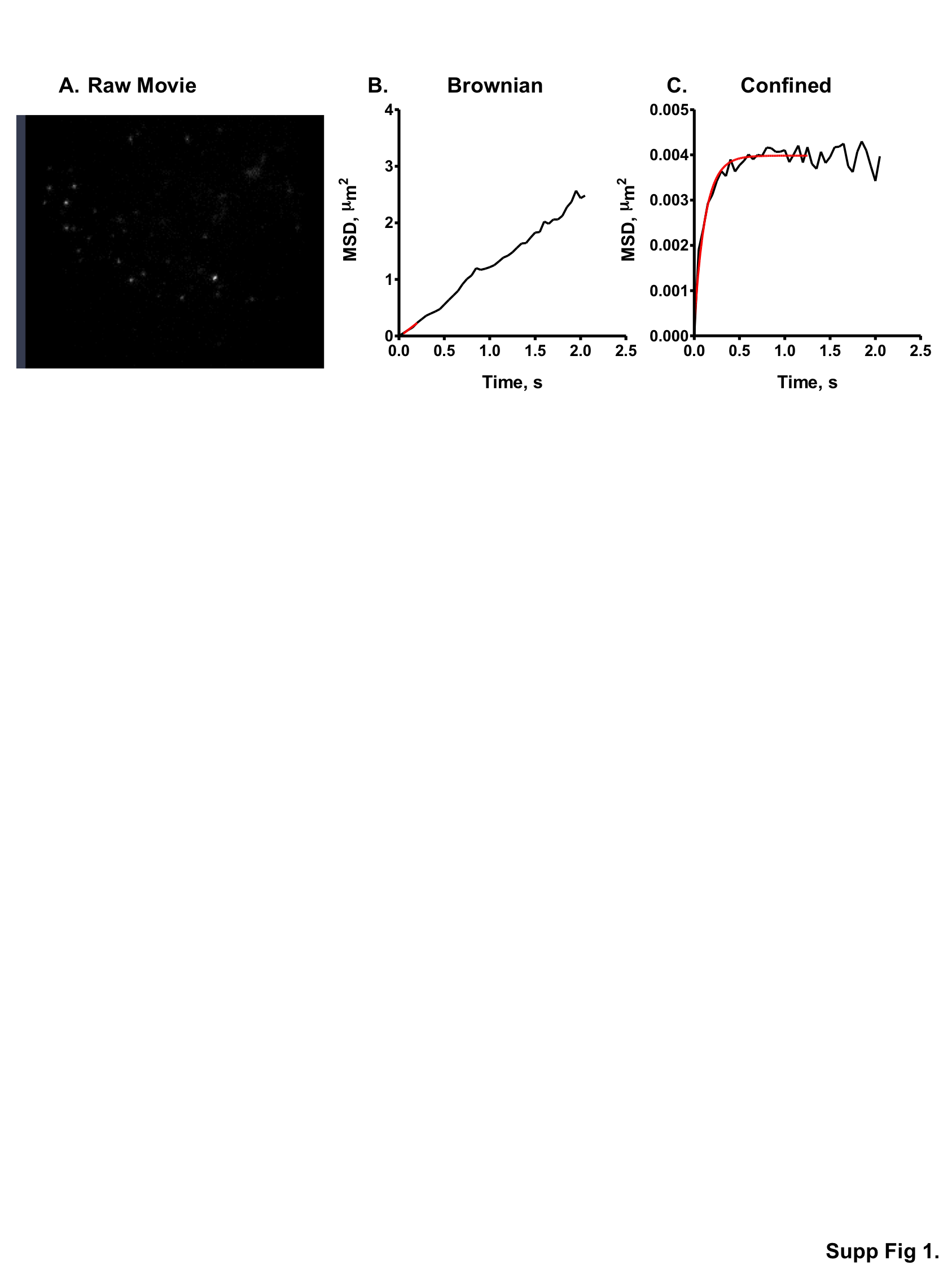

Supplement: Supplementary file 1 [file cmi0015-0430-SD1.zip › SFigure1.tiff]

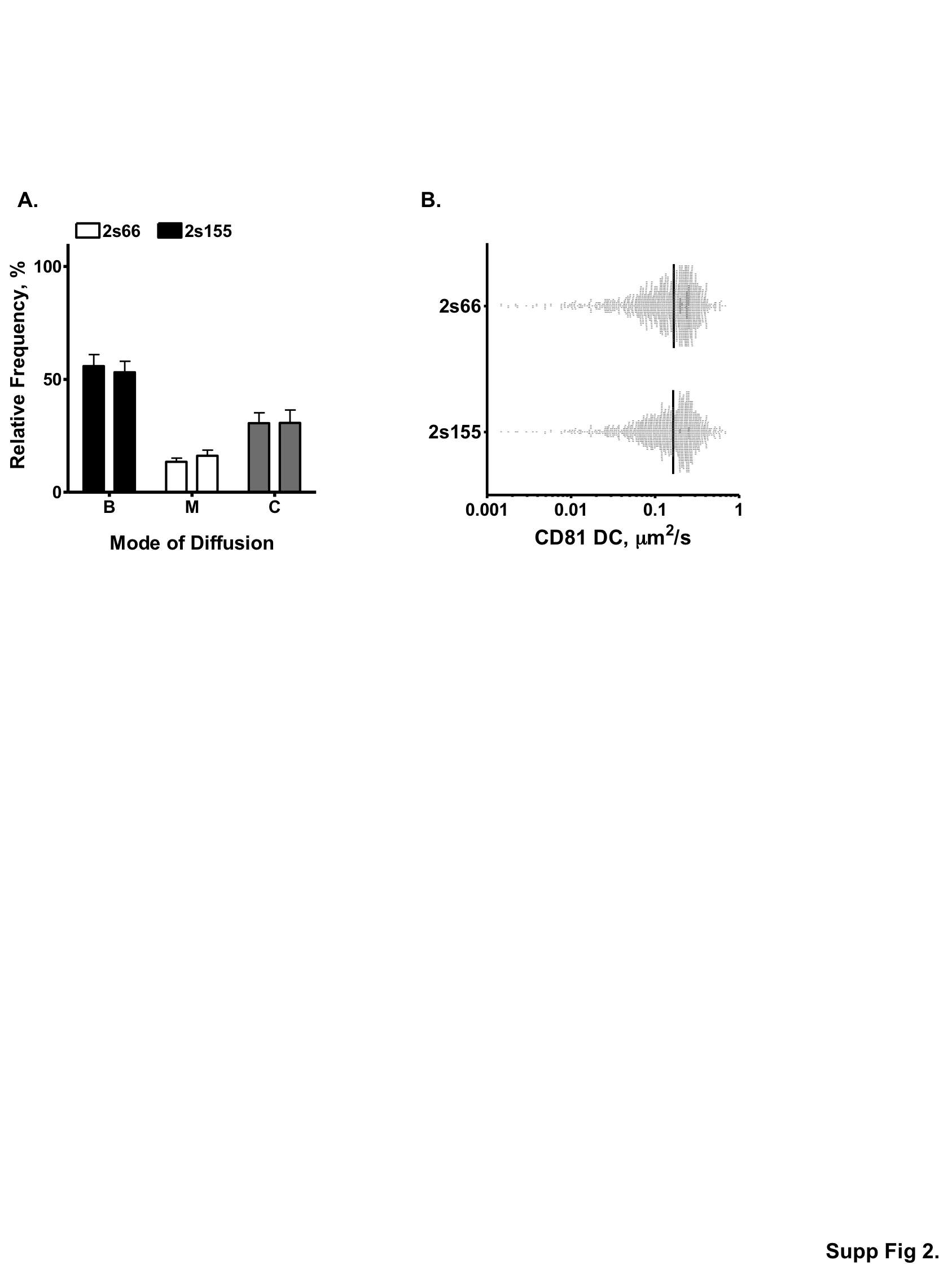

Supplement: Supplementary file 2 [file cmi0015-0430-SD2.tiff]

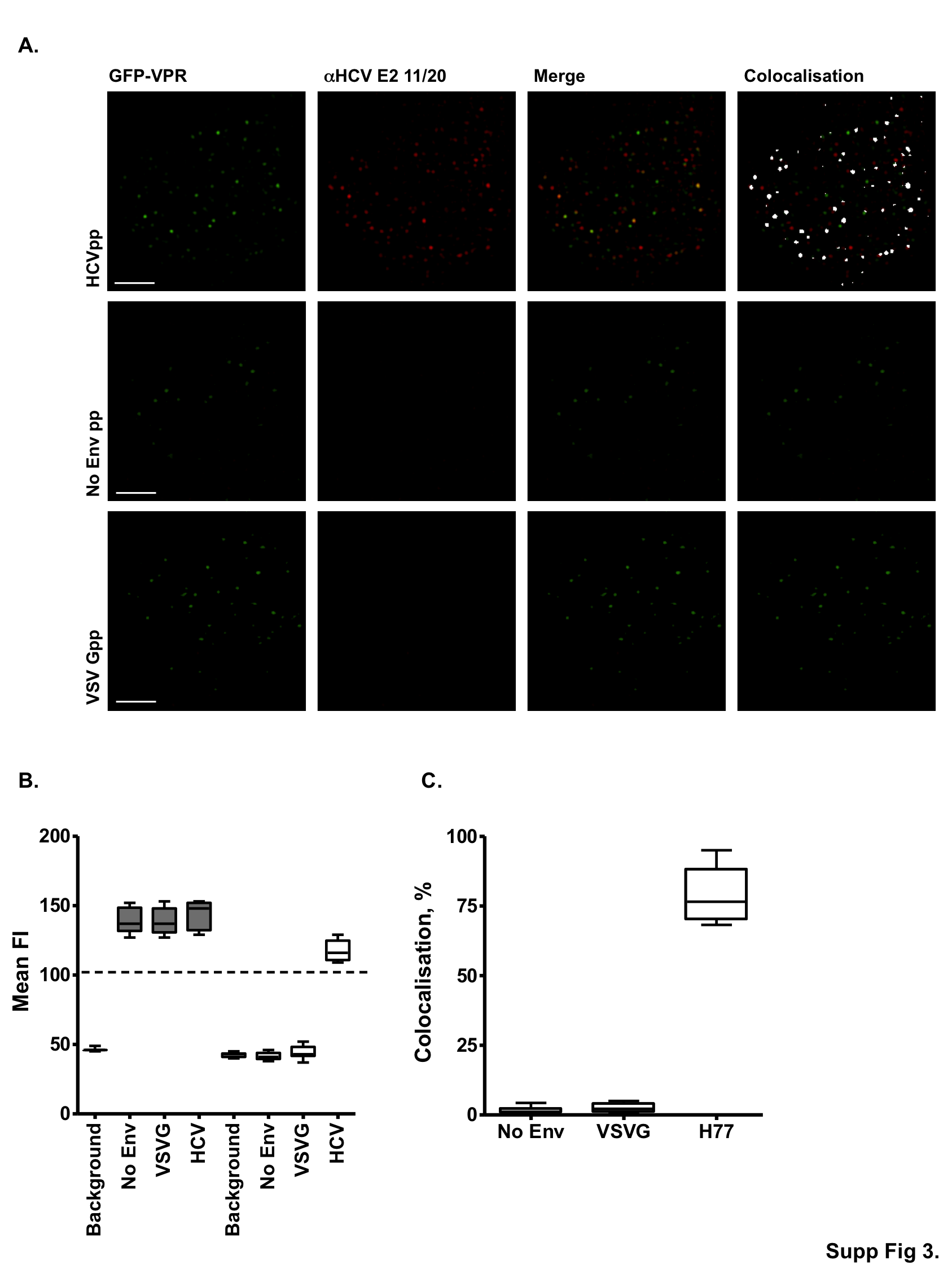

Supplement: Supplementary file 3 [file cmi0015-0430-SD3.tiff]
