# Supplementary material for: Hepatoma polarization limits CD81 and hepatitis C virus dynamics
Source: Cell Microbiol. 2012 Nov 20;15(3):430–45. doi: 10.1111/cmi.12047 (PMC3599488; doi:10.1111/cmi.12047)
Supplement: Supplementary file 1 [file cmi0015-0430-SD1.zip › Revised Supplementary Figure legends-2 .doc]

**Supplementary Figure legends:**

**Fig.S1. MSD plot and movie, Movie of CD81 dynamics and MSD analysis.** Example of CD81 single molecule tracking using Atto647N-labeled Fab fragments (A). A neural network (see Materials and Methods) is used to determine CD81 behavior within a trajectory. Each identified segment is then analysed using MSD analysis and a plot of Brownian (B) and confined (C) segments. The first 4 points are used to determine the apparent diffusion coefficient of the molecule. The fit of the confined trajectory has been performed using 50% of the dots.

**Fig.S2**. **CD81 single molecule tracking profiles.** The relative frequency of CD81 trajectories with Brownian (B), mixed (M) or confined (C) behaviour observed with anti-CD81 Fabs 2s66 and 2s155 (A). A minimum of 10 cells and 250 trajectories were measured per parameter as predetermined by BMC sampling. Scatter plots for Brownian CD81 diffusion coefficients measured with anti-CD81 Fabs 2s66 or 2s155 in non-polarized HepG2 cells, where the median Brownian DC values for 2s66 and 2s155 are 0.17±0.2µm^2^/s and 0.16±0.2µm^2^/s, respectively (B). Each point represents one trajectory with the vertical line indicating the median.

**Fig.S3**. **Anti-HCV E2 specificity**. Representative TIRF images of GFP.VPR HCVpp, Env-pp or VSV Gpp bound to non-polarized HepG2 cells co-stained with αnti-HCV E2 11/20 Fab-Atto647N. 79% of GFP.VPR expressing HCVpp co-stained with αnti-HCV E2 11/20. The threshold for the green and red signals was defined as twice the mean background plus 3 standard deviations of uninfected HepG2 cells. Co-localization was established following the analysis of 40 cells. Scale bar represents 10µm.
